# Measuring and Energizing Sensor System for Digital Signal Monitoring of an Academic–Experimental CubeSat for Wireless Telemetry Purposes

**DOI:** 10.3390/s23198299

**Published:** 2023-10-07

**Authors:** Jose Pablo Garcia-Fernandez, Leobardo Hernandez-Gonzalez, Jazmin Ramirez-Hernandez, Pedro Guevara-Lopez, Oswaldo Ulises Juarez-Sandoval, Guillermo Avalos-Arzate

**Affiliations:** Escuela Superior de Ingenieria Mecanica y Electrica, Unidad Culhuacan, Instituto Politecnico Nacional, Av. Santa Ana No. 1000, Col. San Francisco Culhuacan, Mexico City 04440, Mexico; pablogfdz07@gmail.com (J.P.G.-F.); pguevara@ipn.mx (P.G.-L.); ojuarezs@ipn.mx (O.U.J.-S.); gavalosa@ipn.mx (G.A.-A.)

**Keywords:** CubeSat-1U, telemetry, sensors, energy

## Abstract

Space technology for small satellites has made significant progress in the academic and industrial fields, and an alternative focused on educational institutions is the CubeSat standard, created to promote various scientific projects of space exploration. In this context, a fundamental module of any satellite is the telemetry subsystem, which controls the communication with the Earth through electronic circuits dedicated to remote communication; also, the measurement and power supply modules are integrated into a CubeSat. Its construction costs range from USD 2500 to 55,000, with suppliers from Europe and the United States. This motivates the development of the present project, aimed at an academic–experimental CubeSat-1U prototype, to limit this technological dependence, focusing on the measurement generated by the acceleration sensors, angular velocity, magnetic fields, barometric pressure, temperature and ultraviolet light intensity, and the energization of each of them. For this, the main objective of the research is to identify the four basic subsystems of the CubeSat-1U: (a) energization subsystem, (b) sensing subsystem, (c) transmission and reception subsystem, and (d) control subsystem. To describe in detail the construction of (a) and (b), a set of diagrams is performed, defining their operation and its interaction. To explain the subsystem’s construction, the components selection and integration are presented. As a result, the electrical measurements generated by the power system, the output of the sensors in laboratory conditions, and images of the developed circuits are presented, having as a contribution to the methodology of design, integration, and development of the four subsystems, the feasibility of construction and its implementation in an academic satellite.

## 1. Introduction

The CubeSat standard was developed in collaboration with the California Polytechnic State University, San Luis Obispo, and the Stanford University Laboratory for the Development of Space Systems in 1999 [1,2] to reduce costs and implement pico-satellites to achieve access to space technology in support of university science. Today, CubeSat developers are not only universities and educational institutions but also private companies and government organizations. A CubeSat must meet specific criteria regarding its shape, size, and weight; therefore, the engineering and development of CubeSats becomes less expensive than small, highly customized satellites (less than 300 kg, 1100 lb). In addition, their standardized shape and size reduce the costs associated with shipping and its placement in the space. These satellites are defined in various sizes based on the standard CubeSat “unit”; a 10 cm cube per side with a mass between 1 kg and 1.33 kg is denoted as 1U [3]. 

In [4], the CubeSat CO-57 (XI-IV) developed in 2000 was the first handmade prototype developed by ISSL (Intelligent Space Systems Laboratory) at the University of Tokyo (UT). The prototype stabilizes on its three axes; the altitude is controlled by a permanent magnet; the power is provided by six solar cells and a lithium-ion manganese battery of 780 mAh. In [5], the educational satellite SATEDU was designed and manufactured at the Engineering Institute of the Universidad Nacional Autónoma de México in 2009. It has several subsystems: power supply, flight computer, wireless communications, satellite platform sensors, inertial wheel stabilization, and inertial navigation sensors. Its average power consumption is 500 mA, with four lithium-ion batteries from 3.7 V at 890 mAh. A computer emulates an Earth station that makes real-time 3D tracking of the CubeSat movements. In [6,7], the 3U-CubeSat STRaND-1 (Surrey Training Research and Nanosatellite Demonstration-1) was developed by the Surrey Space Center of the University of Surrey, U.K., in collaboration with Surrey Satellite Technology Ltd. (SSTL) and put into orbit by an Indian PSLV C-20 space shuttle in 2013. It works using an Android operating system, so it is considered the first phone-sat in the world. The smartphone integrated into the CubeSat provides cameras, radio links, accelerometers, and high-performance processors. In [8], the international development of CubeSats is shown. The investigations are led by the United States of America and Russia, followed by India and China. Significant progress in this area is lacking in Latin America.

The telemetry module is fundamental in the CubeSat design. It is based on the conversion of captured signals by a set of sensors to electrical signals that are transmitted to an Earth station. The monitored signals are vibrations, temperature, pressure, voltage, etc [9]. In this paper, the acquisition, analysis, and integration of the digital signals from different sensors in a CubeSat are proposed.

The rest of the paper is organized as follows: in Section 2, the electronic structure of the academic–experimental CubeSat is presented; in Section 3, the subsystems design methodology and a brief description is shown. In Section 4, the experimental prototype development and the experimental results are described; finally, in Section 5, the conclusions are presented.

## 2. CubeSat Electronic Structure 

**Figure 1:**
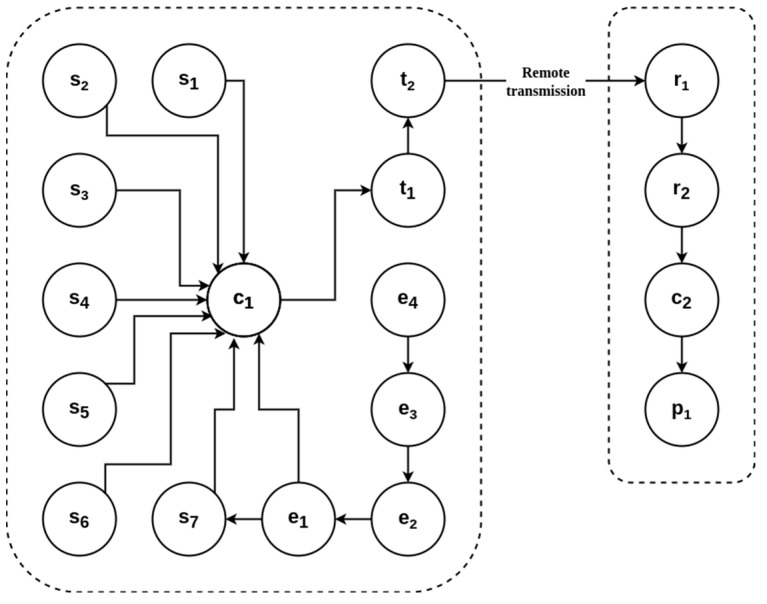
Telemetry sets diagram for the CubeSat integration.

For the CubeSat development, four basic subsystems that are shown in Figure 1 have been analyzed and designed, such as: ∑ = {∑_1_, ∑_2_, ∑_3_, ∑_4_}

∑_1_ is the energization set: ∑_1_ = {e_1_, e_2_, e_3_, e_4_}
∑_2_ is the sensing set: ∑_2_ = {s_1_, s_2_, s_3_, s_4_, s_5_, s_6_, s_7_}
∑_3_ is the transmission and reception set: ∑_3_ = {t_1_, t_2_, r_1_, r_2_}
∑_4_ is the control set: ∑_4_ = {c_1_, c_2_, p_1_}

Energization set: ∑_1_ = {e_1_, e_2_, e_3_, e_4_}

e_1_: Voltage booster
e_2_: Battery charger
e_3_: DC/DC buck converter
e_4_: Solar panels

Sensing set: ∑_2_ = {s_1_, s_2_, s_3_, s_4_, s_5_, s_6_, s_7_}

s_1_: Acceleration
s_2_: Angular velocity
s_3_: Magnetic field
s_4_: Temperature
s_5_: Pressure
s_6_: Ultraviolet light
s_7_: Battery charge level

Transmission and reception set: ∑_3_ = {t_1_, t_2_, r_1_, r_2_}

t_1_: Transmitter
t_2_: Antenna
r_1_: Receiver
r_2_: Antenna

Control set: ∑_4_ = {c_1_, c_2_, p_1_}

c_1_: Remote computer
c_2_: Local computer
p_1_: Ground terminal

1. ∑_1_ provides electrical power to the CubeSat subsystems; this power is obtained from the set of five panels. The voltage provided by the panels (e_4_) is filtered and regulated by a DC/DC buck converter (e_3_), which is used as a battery charger (e_2_) and voltage booster (e_1_). Note that e_1_ feeds directly to the remote computer c_1_, while e_2_, e_3,_ and e_4_ do not because the power supply is not ready yet.
2. ∑_2_ measures physical parameters, such as acceleration (s_1_), angular velocity (s_2_), magnetic field (s_3_), temperature (s_4_), pressure (s_5_), ultraviolet light intensity (s_6_), and battery charge level (s_7_).
3. ∑_3_ is used to transmit information obtained by sensors via radio frequency (RF) {t_1_, t_2_} to the ground terminal complex for its analysis and processing {r_1_, r_2_, p_1_}.
4. ∑_4_ manages and processes each algorithm included in the previously mentioned subsystems {c_1_, c_2_}. The final information must be transmitted to the ground terminal complex and presented by p_1_.

## 3. Subsystems Interaction and Operation

**Figure 2:**
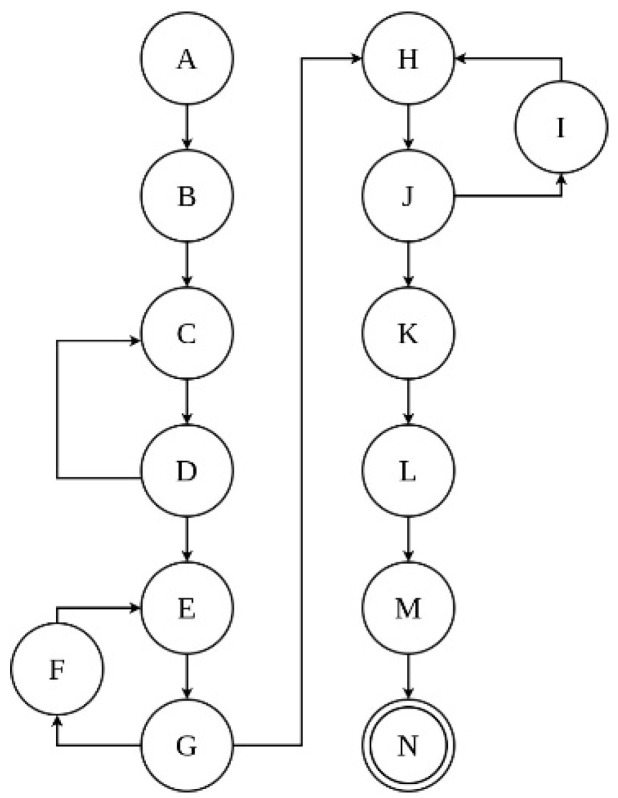
General state diagram of the telemetric system.

The electronic interaction and operation of the CubeSat subsystems can be represented by a state diagram, as is shown in Figure 2, where each subsystem operation is described.

Figure 2 is composed of 14 states that, in combination, represent the set ∑ = {A, B, C, D, E, F, G, H, I, J. K, L, M, N}, where:

- A: Start
- B: Circuit power
- C: Regulation and battery charging
- D: Check of the battery charge
- E: Arduino nano energization and sensors activation (accelerometer, gyroscope, magnetometer, temperature, barometer, UV intensity, and battery charge level)
- F: Sensors reset
- G: Reception of information from sensors
- H: Transmission of signals via RF to the Earth station
- I: Make a new reading of the device.
- J: CubeSat RF signal reception checking
- K: Processing and graphing of the CubeSat signals
- L: Data storage for comparison
- M: Results
- N: End

### 3.1. Design of the Energy System

**Figure 3:**
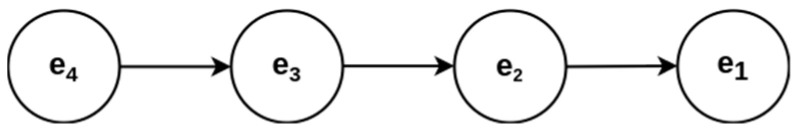
Block diagram of the energy subsystem.

The set diagram of the CubeSat’s power subsystem (∑_1_) is shown in Figure 3. The energy to provide the voltage and current requirements of the entire electronics system is obtained from the panels (e_4_) and is filtered and regulated by a DC voltage regulator Buck converter (e_3_), which is used as a battery charger (e_2_) and voltage booster (e_1_). Table 1 lists the voltage and current requirements for all components.

**Figure 4:**
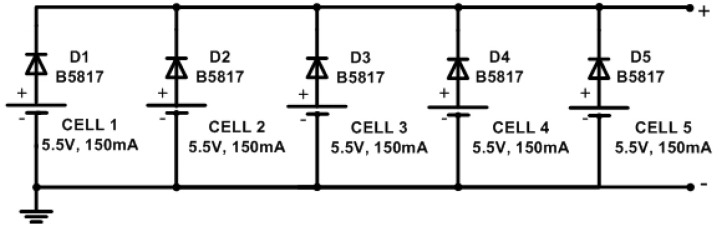
Solar panels array for energization.

To achieve a greater energy collection during the day, an array of five solar panels is used, as shown in Figure 4. The array allows for obtaining a nominal voltage of 5.05 V at 750 mA.

**Figure 5:**
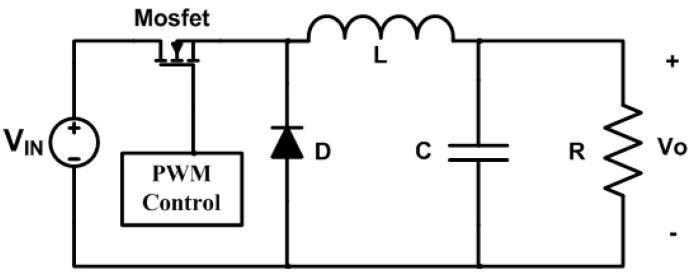
Buck converter scheme.

At the output of the solar panel array, a buck converter is integrated to regulate the 4.2 V needed to charge the lithium battery in the backup module. The basic scheme is shown in Figure 5. The design of the buck converter uses the following data: *V_in_* = 5.5 V, *V_o_* = 4.2 V, *V_d_* = 1 V, *I_o_* = 150 mA, *F* = 150 kHz. ∆*I* = 30% of *I_o_*, and ∆*V_o_* = 5% of *V_o_*. [10].

(1) $$D=\frac{V_{o}+V_{d}}{V_{in}}=\frac{4.2\;V+1\;V}{5.5\;V}=0.94$$

(2) $$L=\frac{V_{o}\left(1-D\right)}{\Delta I*F}=\frac{4.2\;V\left(1-0.94\right)}{0.3\left(150\mathrm{mA}\right)\left(150\mathrm{kHz}\right)}=46.2\;\mu H$$

(3) $$C=\frac{V_{o}\left(1-D\right)}{8\left(\Delta V_{0}\right)\left(L\right){\left(F\right)}^{2}}=\frac{4.2\;V\left(1-0.94\right)}{8\left(30\mathrm{mV}\right)\left(46.2\;\mu H\right){\left(150\mathrm{kHz}\right)}^{2}}=1.25\;\mu F$$

The commercial values of the inductor and capacitor are: *L* = 47 μH and *C* = 2.2 μF.

To ensure the operation of the power supply despite variations in the solar panels, a Lipo battery of 3.7 V at 2800 mAh is used. The total current demand, *I_tot_*, in the circuit is the sum of the currents of all devices, according to (Equation (4)).

(4) $$I_{tot}=I_{Ardu}+I_{accel}+I_{pres}+I_{gyro}+I_{magn}+I_{ina}+I_{UV}+I_{HC}=362\;\mathrm{mA}$$

where *I_Ardu_, I_accel_, I_pres_*, *I_gyro_*, *I_magn_*, *I_INA_*, *I_UV_*, and *I_HC_* are the current demands in the microcontroller, accelerometer sensor, pressure sensor, gyroscope sensor, magnetometer, current sensor, UV intensity sensor, and transmitter, respectively. The sensor’s total current consumption is 362 mA, less than the 750 mA that can be supplied by the solar panel array, ensuring adequate supply under nominal operating conditions. 

The battery backup time at full charge, for a consumption of 362 mA is indicated in (Equation (5)),

(5) $$T_{\sup}=\frac{V_{bat}I_{bat}}{V_{tot}I_{tot}}=\frac{\left(3.7\;V)(2800\;\mathrm{mA}\times h\right)}{\left(5\;V)(362.152\;\mathrm{mA}\right)}=5.72\;h$$

where *V_bat_* and *I_bat_* are the nominal battery voltage and current capacity, indicating the amount of current that the battery can supply over time. *V_tot_* and *I_tot_* are the total voltage and current required by the circuit.

According to (Equation (2)), the battery can supply voltage for approximately five hours and forty-three minutes. A backup time of 6 h is considered sufficient for the proposed objective. In this critical condition, the proposed system would work only in sensing mode and would not send telemetry information to the ground station, increasing the backup time to about 12 h by reducing the 200 mA consumption of the HC-12 transmitter. 

**Figure 6:**
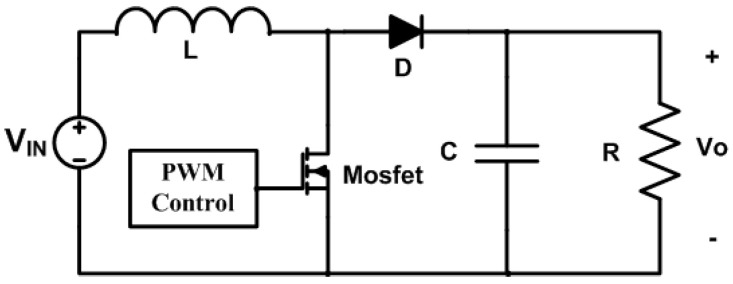
Boost converter scheme.

The battery voltage drops from 4.2 V to a minimum of 3.7 V. This voltage is below the 5 V required for the control system energization; therefore, a booster circuit is required to keep the output voltage constant. A typical boost topology is shown in Figure 6. 

The design of the boost converter uses the following data: *V_in_* = 3.7 V, *V_o_* = 5 V, *I_o_* = 362 mA, *F* = 400 kHz. ∆*I* = 20% of *I_o_*, and ∆*V_o_* = 5% of *V_o_*. [10].

(6) $$D=1-\frac{V_{in}}{V_{o}}=\frac{3.2\;V}{5\;V}=0.36$$

(7) $$L=\frac{\left(V_{in}\right)\left(D\right)}{\left(\Delta I\right)\left(f_{s}\right)}=\frac{3.7\;V\left(0.36\right)}{0.2\left(362\mathrm{mA}\right)\left(400\mathrm{kHz}\right)}=46.25\;\mu H$$

(8) $$C=\frac{\left(D\right)\left(Io\right)}{\left(\Delta V_{0}\right)\left(f_{s}\right)}=\frac{0.36\left(362\mathrm{mA}\right)}{\left(50\mathrm{mV}\right)\left(400\mathrm{kHz}\right)}=6.48\;\mu F$$

The commercial values of the inductor and capacitor are *L* = 47 μH and *C* = 10 μF.

The designed buck converter stabilizes the voltage supplied by the solar cell array to power the backup battery and the ADXL345, BMP085, L3G4200D, and HMC5883L sensors. The boost converter supplies the power to the Atmega328P microcontroller, the INA226 current sensor, and the HC-12 transmitter.

### 3.2. Sensing and Control Subsystems Design

**Figure 7:**
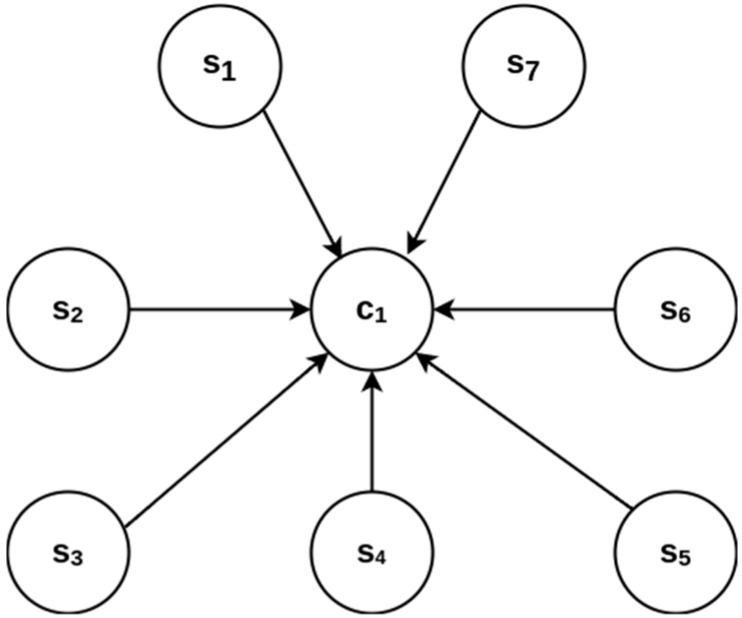
Sensing and control subsystems block diagram.

The sensing and control subsystems (∑2) are combined in the block diagram shown in Figure 7, where the sensing subsystem is composed of the integration of seven sensors in combination with the control subsystem integrated into the CubeSat, such as the ATMEGA328P microcontroller. The sensing subsystem starts its development with the correct configuration of each proposed sensor, and the control subsystem starts its development with the correct configuration of the I²C communication protocol to manage the data received from the sensors. Remember that s_1_ measures acceleration, s_2_ measures angular velocity, s_3_ measures magnetic field, s_4_ measures temperature, s_5_ measures pressure, s6 measures ultraviolet light intensity, and s_7_ measures battery charge level.

The selected parameters for the correct configuration of each sensor are described as follows. 

#### 3.2.1. Voltage and Current Sensor for the Backup Energy

The proposed CubeSat development is designed with the incorporation of a source energy backup that, according to the requirements, is a battery. To evaluate the charging level of the battery, the voltage and current are sensed by the INA226 sensor that is configured by the INA226.h library, and the voltage and current variables must be declared as floating point. The sensor is calibrated to be able to sense 1 A as the maximum expected current through a shunt resistance (*R_shunt_*) with a value of 0.1 Ω. According to the sensor specification [11], it senses the differential voltage between the positive and negative input terminals (IN+, IN−) and the VBUS terminal. Once the differential voltage between the *R_shunt_* terminals is obtained, the current and power are calculated by the listed process in Table 2.

The input range of the bus voltage is 0 to 36 V, which is represented by 40.96 V on the ADC scale. Finally, in step 5, the power is obtained according to the datasheet of the microcontroller [11].

In step 4, the bus voltage is 3920. This value is obtained by (Equation (3)), where *V_derivation_* = 4.9 V. Resolution_ADC = 32,768 and *V_max_* = 40.96. Finally, in step 5, the power is obtained according to the microcontroller datasheet [11].

(9) $$\begin{array}{l} V_{bus}=\mathrm{size}\text{\_}\mathrm{LSB}\;\times \;\mathrm{Resolucion}\text{\_}\mathrm{ADC}=1.25\;\mathrm{mV}\;\times 2^{15}=40.96\;V\\ Bus\;Voltage=\frac{V_{derivation}\times \;\mathrm{Resolution}\_\mathrm{ADC}}{V_{max}} \end{array}$$

#### 3.2.2. Telemetry Sensors

The CubeSat design considers the integration of a sensing subsystem used for weather applications; according to Figure 7, each block represents a device that measures a specific parameter; however, to optimize the space and the weight, is considered the use of the inertial measurement units GY-80 (IMU GY-80), that integrates the L3G4200D 3-axis gyroscope used as angular velocity sensor [12], ADXL345 3-axis accelerometer used as acceleration sensor [13], HMC5883L 3-axis magnetometer used as magnetic field sensor [14], BMP085 barometer/thermometer used as pressure and temperature sensor [15]. To quantify the UV intensity is proposed the ML8511 sensor [16], which is not integrated into the GY-80. 

According to the IMU GY-80, the data communication of each sensor is based on the I²C protocol. In Table 3 are defined the registers used to initialize each sensor. 

Considering that the microcontroller ATMEGA328P resolution is 10 bits and the reference voltage of the ML8511 is 3.3 V, the minimum step to be sensed for the UV sensor is calculated by (Equation (10)),

(10) $$Minimum\;step=\frac{V_{ref}}{2^{n}-1}=\frac{3.3\;V}{2^{10}-1}=3.22\;\mathrm{mV}$$

where the *V_ref_* is the reference voltage and *n* is the microcontroller resolution. Finally, the UV intensity is obtained by Equations (11) and (12).

(11) $$V_{sensed}=\frac{\left(V_{pin\_\mathrm{analog}}\right)\left(V_{ref}\right)}{2^{n}-1}$$

(12) $$UV\;\;Intensity=\frac{V_{sensed}}{Minimum\;step}$$

where *V_pin_analog_* is defined as the voltage measured by the microcontroller’s ADC output.

### 3.3. Transmission and Reception System and Control System Design

**Figure 8:**
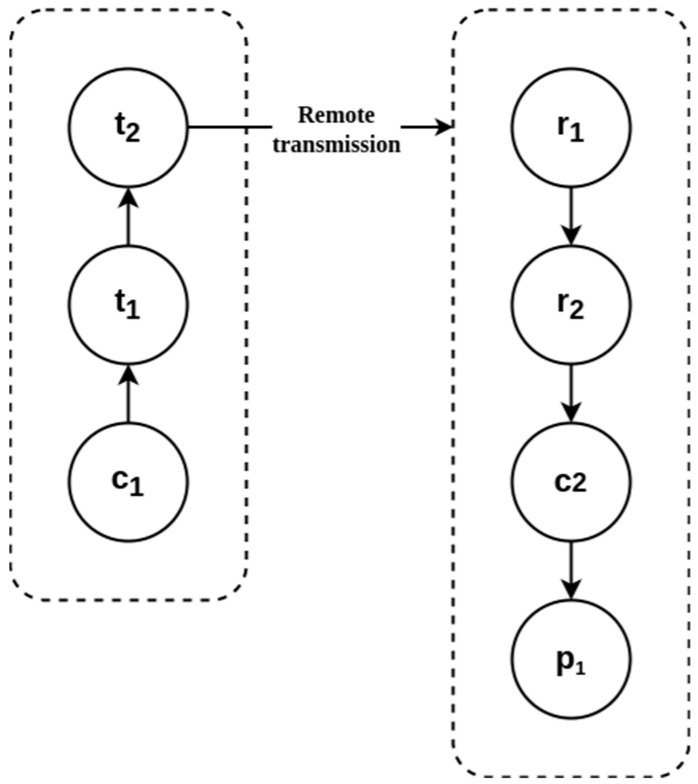
Set diagram of transmission and reception subsystem.

The set diagram of the transmission and reception subsystems (∑3) implemented in the CubeSat is shown in Figure 8. It is used to transmit information obtained by sensors via radio frequency (RF) {t_1_, t_2_} to the ground terminal complex for its analysis and processing {r_1_, r_2_, p_1_}. c_1_ and c_2_ are the microcontrollers Arduino nano remote and local, respectively, and p_1_ is a local computer used to receive and process data of telemetry. In this section, ∑_4_ is the control system; it is composed of a local computer c_2_, a remote computer c_1,_ and a ground terminal p_1_.

#### HC-12 Transmitter Configuration

The configuration of this module was achieved with serial communication UART. Using the serial monitor of the microcontroller, AT commands are programmed to allow preset configurations. Table 4 shows the description of each command.

The transmitter module is configured with the AT + B9600, AT + CH001, AT + FU3, and AT + P8 commands, which configure the transmission module as follows: transmission rate at 9600 Bauds, transmission channel 1 to 443.4 MHz, transmission of 8-bit data + 1 parity bit. To achieve simplex communication, the same commands are configured in the receiving module. The information obtained from the sensors is sent by the serial port every 250 ms to the receiver to be displayed in the graphical interface.

## 4. Results

In this section, the tests performed for each module that integrates the CubeSat are presented. 

### 4.1. Power System

**Figure 9:**
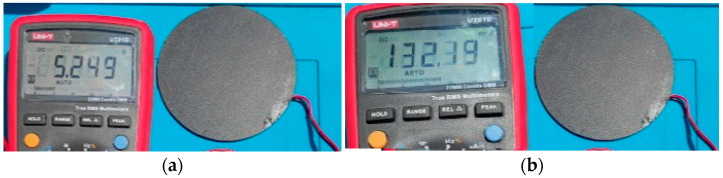
Voltage and current provided by the solar panels: (**a**) voltage 5.24 V and (**b**) current 132.19 mA, with an output load of 0.82 Ω.

The voltage and current measurements provided by the solar panel with a 0.82 Ω resistive load are shown in Table 5 and Figure 9. The values obtained are lower than the data provided by the manufacturer; therefore, the measured data are used for design purposes to quantify the power provided by the solar panel to the system. It is determined by environmental conditions (such as temperature, shading, and dust accumulation), degradation, or aging of the solar panel. Measurement inaccuracies can occur due to equipment calibration limitations or variations in the resistive load during measurements.

**Figure 10:**
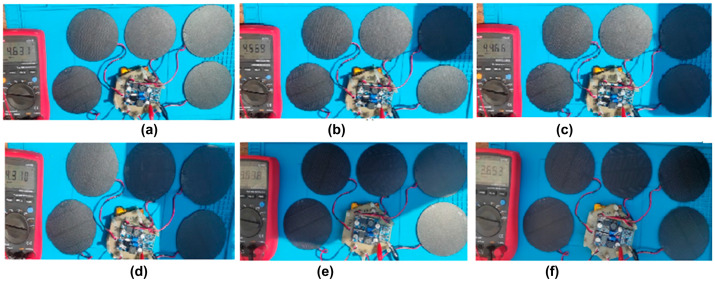
Output voltage in the Buck converter. (**a**) No solar panels covered by shadow, 4.6 V; (**b**) one solar panel covered by a shadow, 4.5 V; (**c**) two solar panels covered by a shadow, 4.4 V; (**d**) three solar panels covered by a shadow, 4.3 V; (**e**) four solar panels covered by a shadow, 3.6 V; (**f**) all solar panels covered by a shadow, 3.6 V.

The output voltage in the buck converter, considering the solar panels covered by a shadow, is shown in Figure 10. In Table 6, the voltages obtained for each test are listed.

**Figure 11:**
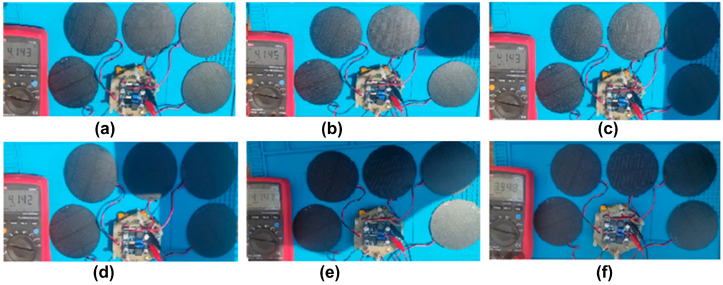
Battery output voltage. (**a**) No solar panels covered by shadow, 4.1 V; (**b**) one solar panel covered by a shadow, 4.1 V; (**c**) two solar panels covered by a shadow, 4.1 V; (**d**) three solar panels covered by a shadow, 4.1 V; (**e**) four solar panels covered by a shadow, 4.1 V; (**f**) all solar panels covered by a shadow, 3.9 V.

The battery voltage that energizes the boost converter is shown in Figure 11; in Table 7, the results obtained are listed.

The test shown in Figure 10 and Figure 11 allows the evaluation of different scenarios where the solar cells do not receive perpendicular sunlight due to the rotation or oscillation of the CubeSat. In addition, its effect on the charge of the backup battery can be analyzed.

### 4.2. Sensor Control System

#### 4.2.1. Communication Protocol

**Figure 12:**
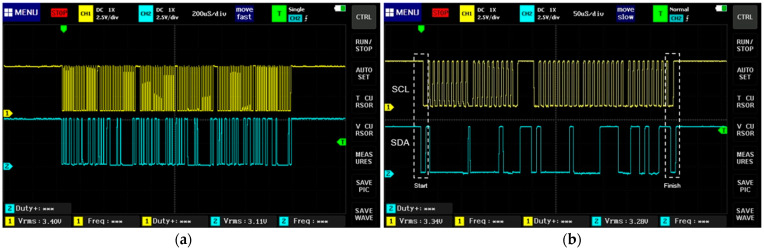
Communication protocol I^2^C; (**a**) SCL and SDA signals; (**b**) start and end conditions.

The communication protocol I^2^C is shown in Figure 12a (the yellow signal is the clock signal SCL, and the blue signal is the data signal SDA). This protocol allows the communication and data reception of the sensors: accelerometer, gyroscope, digital compass, barometer, and current sensor. The communication protocol incorporates a start condition for the SDA signal to start prior to SCL and a stop condition for the SCL signal to end prior to the SDA signal, as is shown in Figure 12b. 

**Figure 13:**
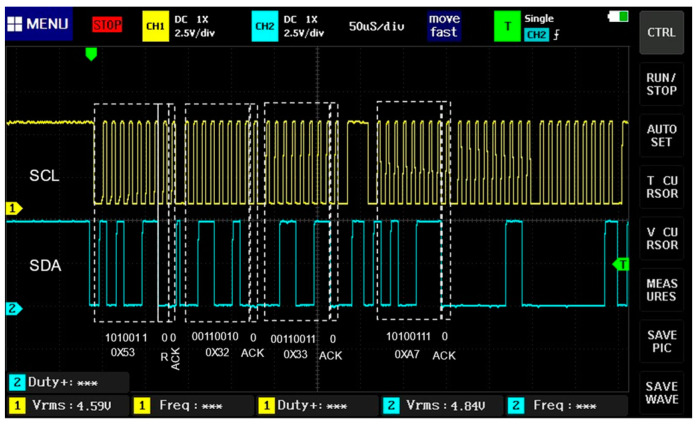
Four frames transmission using I2C protocol.

In Figure 13, a data signal (SDA) of four bit-frames is shown; in the first frame, the initial seven bits are the sensor direction 0 × 53 (accelerometer), and the eighth bit is at a low logical level, indicating that the sensor is in reading mode and the ninth bit (ACK) is at a low logical level, indicating sensor recognition. In the second frame, the first eight bits are the first data sent by the sensor (0 × 32). In the third frame, the first eight bits are the first data sent by the sensor (0 × 33); in the fourth frame, the first eight bits are the first data sent by the sensor (0 × A7); in all the frames, the ninth bit (ACK) is at a low logical level, indicating sensor recognition.

#### 4.2.2. Accelerometer (ADXL354) Tests and Results

**Figure 14:**
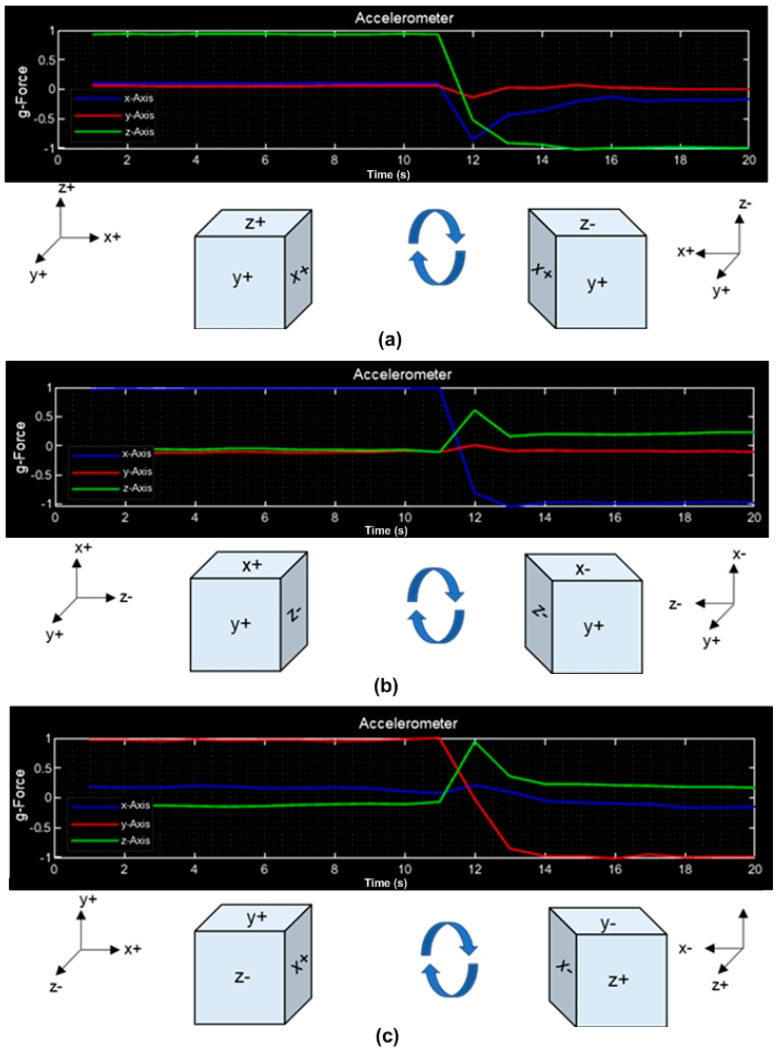
Accelerometer test: (**a**) CubeSat positioned in Z+; (**b**) CubeSat positioned in X+; (**c**) CubeSat positioned in Y+.

Motion and acceleration measurements were made in the three dimensions (X, Y, Z). Figure 14a shows the graph of the accelerometer with the CubeSat positioned in the vertical Z+ axis. After 11 s, the graph is inverted due to the CubeSat being rotated 360° in the vertical axis. Figure 14b shows the graph of the accelerometer with the CubeSat positioned on the X+ axis vertically, then it is inverted 360°. In Figure 14c, the CubeSat is oriented in the Y+ axis, then it is inverted 360°.

**Figure 15:**
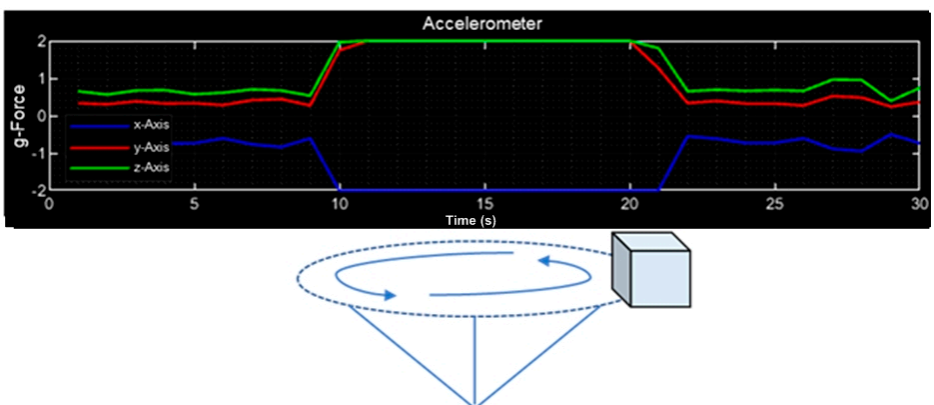
Acceleration measure of the CubeSat.

To measure the acceleration of the CubeSat, it was rotated as is shown in Figure 15, and an acceleration of 2G was obtained, which is equivalent to 19.62 m/s or 70.63 km/h.

The following experimental results validate the entire experimental process and fulfill one of the contributions of this section by validating each of the sensors integrated into the CubeSat.

#### 4.2.3. Gyroscope (L3G4200D) Tests and Results 

**Figure 16:**
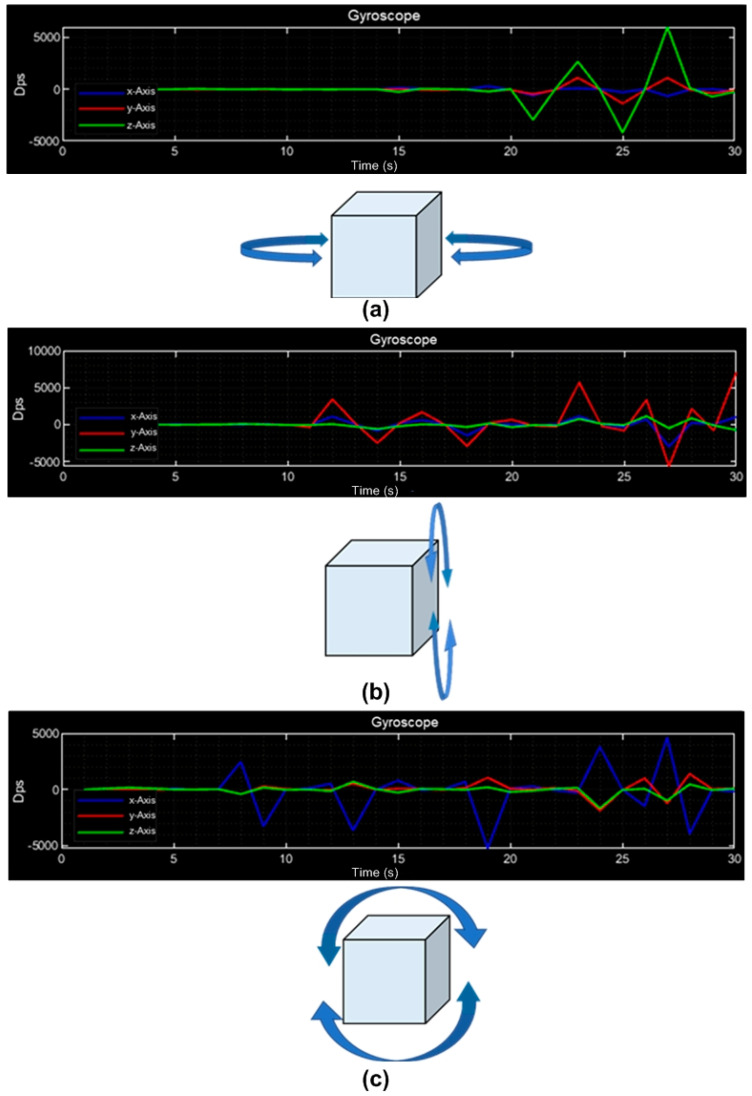
Gyroscope sensed signals; (**a**) in yaw rotation; (**b**) pitch rotation; (**c**) roll rotation.

The signals sensed by the gyroscope are shown in Figure 16. In Figure 16a, the signal of the Z-axis (green) remains constant due to the CubeSat being stationary, then it rotates (yaw movement) on this axis; to the left side, the signal response is negative, and to the right side, the signal is positive. The signals when the CubeSat has a pitch rotation are shown in Figure 16b in red; when the movement is forward, the signal response is positive, and backward, the signal response is negative. The signals when the CubeSat has a roll rotation are shown in Figure 16c in blue; to the left side, the signal is positive, and to the right side, the signal is negative.

#### 4.2.4. Magnetometer (HMC5883L) Tests and Results 

**Figure 17:**
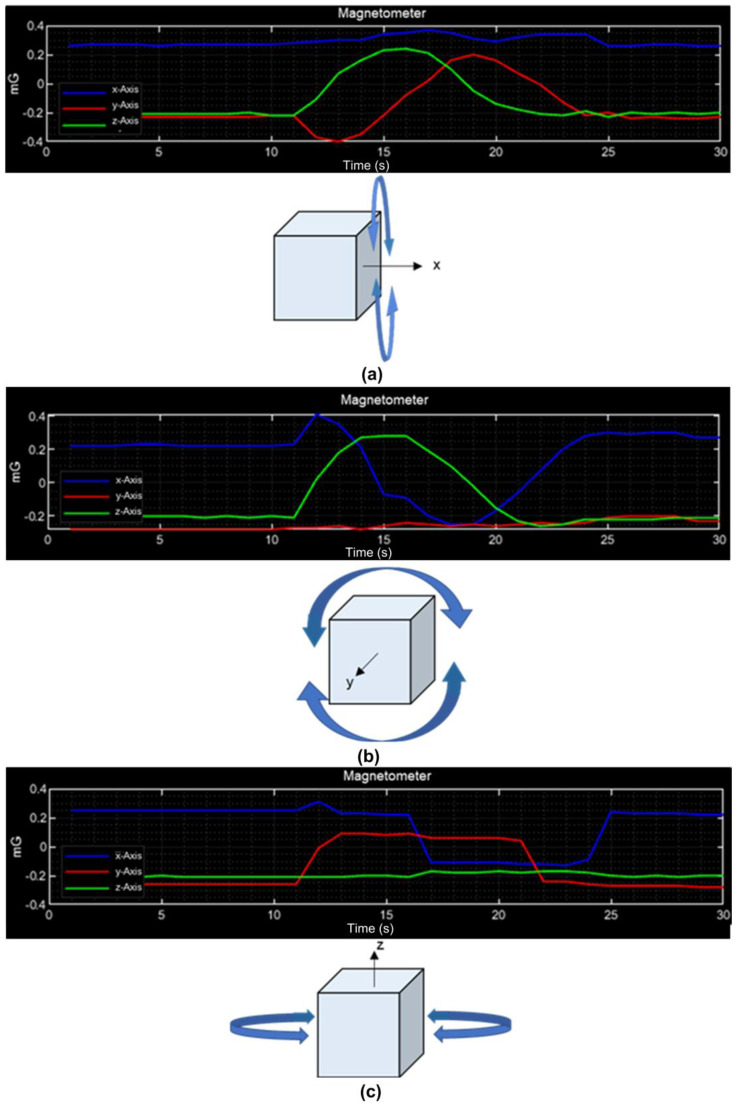
Magnetometer measurements: (**a**) movement on the X-axis; (**b**) movement on the Y-axis; (**c**) movement on the Z-axis.

The behavior of the magnetometer on the three axes is shown in Figure 17; in Figure 17a, initially, the three signals are constant because there is no movement in the CubeSat, and then it is rotated on the X-axis. It is observed that the signals of the Y- and Z-axes change (green and red) while the X-axis signal (blue) presents minimal disturbances. In Figure 17b, the signals of the X- and Z-axes change while the signal of the Y-axis is constant as the rotation is on this axis. In Figure 17c, there is no movement on the Z-axis, so the value remains with minimal changes, while the signals of the X- and Y-axes change.

**Figure 18:**
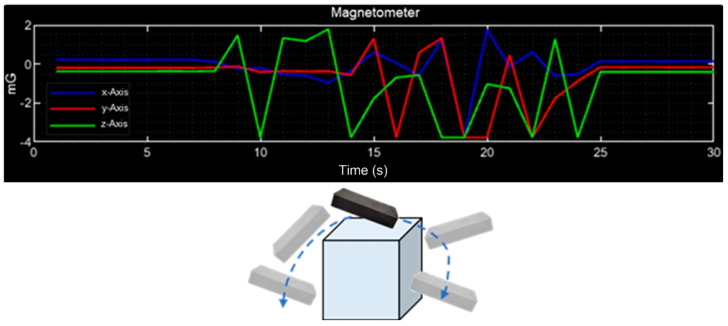
Magnetometer with magnetic disturbance.

The response of magnetometer signals when the CubeSat is exposed to a small magnet is shown in Figure 18. It is observed that by placing the magnet above the device, the reading is altered on the Z-axis (green), repelling the magnet in the time interval 10–15 s; in the interval 15–25 s, signal disturbances are observed when the magnet is placed around the device.

#### 4.2.5. Temperature and Barometer Sensing (BMP085) Test and Results

**Figure 19:**
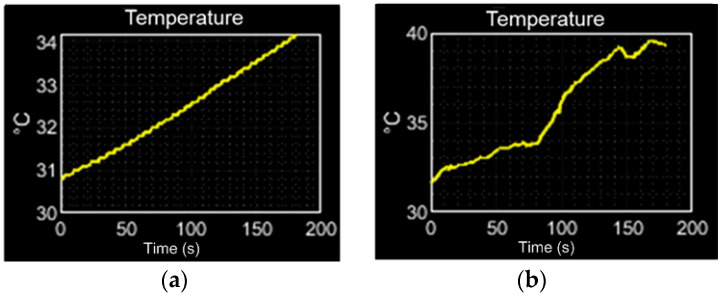
Temperature performance evaluation, (**a**) linear response curve, (**b**) non-linear response curve.

To evaluate the performance of the temperature sensor, linear and non-linear responses are considered. The linear response is evaluated by the exposure of the BMP085 sensor to a controlled temperature supply, such as an incandescent lA bulb, as is shown in Figure 19a. The obtained response remains linear. To evaluate the performance of the temperature sensor with a non-linear response, the sensor is exposed to the sunlight, considered as a variable temperature supply. In this case, Figure 19b shows the irregularity of the response curve that corresponds to the cloud interference.

**Figure 20:**
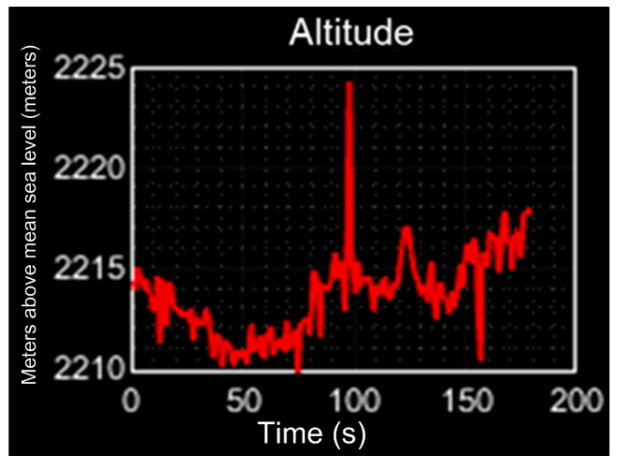
Altitude performance evaluation.

Mexico City is located 2240 m over the sea level; the performance evaluation of the altitude measured is shown in Figure 20, with a value in the range of 2215 m ≤ altitude ≤ 2223; the error precision (*E_p_*) obtained is 0.75% ≤ *E_p_* ≤ 1.1%. 

**Figure 21:**
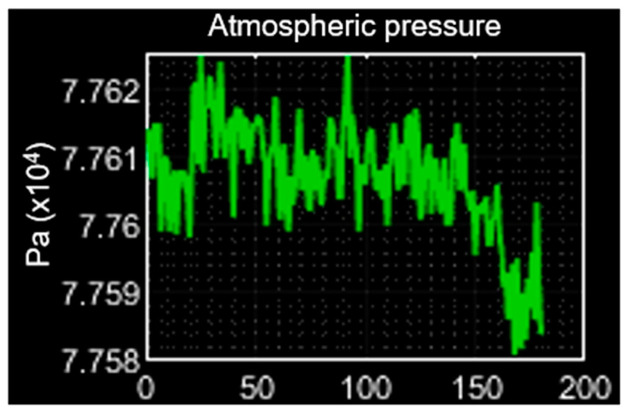
Pression performance evaluation.

According to the governmental dependence of Mexico City named CONAGUA, the pression is located in the range of 775.3 hPa ≤ pression ≤ 779.1 hPa [17], and its average is 777.2 hPa. In comparison with the average value of 776.1 hPa obtained from Figure 21, the pression sensing evaluation shows a better performance.

#### 4.2.6. Ultraviolet (ML8511) Test and Results

**Figure 22:**
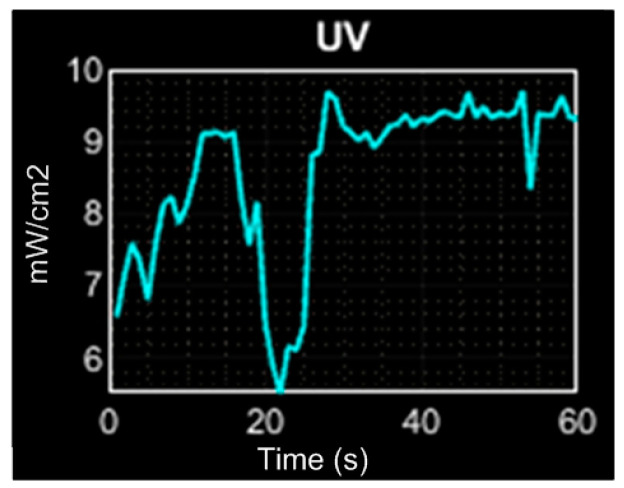
UV light performance evaluation.

To evaluate the UV sensing performance, the proposed ML8511 sensor is exposed to sunlight, and the data are compared with the weather online report of METEORED [18] at the moment of the experimental test. As is shown in Figure 22, the UV sensor shows the UV light obtaining an average value of 9.7 mW/cm^2^, which, in comparison with the online report of 9.55 mW/cm^2^, shows similar results. To observe a variation in the UV light, the sunlight is obstructed, and its variation can be perceived in t = 20.

#### 4.2.7. Voltage and Current Sensor (INA226) Tests and Results

**Figure 23:**
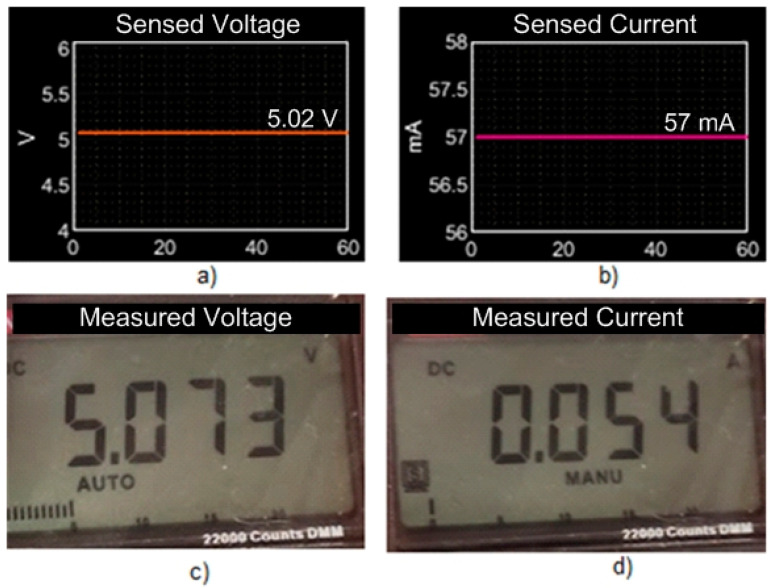
Voltage and current measurements; (**a**) sensed voltage; (**b**) sensed current; (**c**) measured voltage; (**d**) measured current.

The voltage and current values measured by the INA226 sensor are shown in Figure 23a,b; in the same figure are contrasted the values of voltage and current measured with a multimeter. It can be seen that the values are approximated.

#### 4.2.8. Transmission and Reception Systems Tests and Results

**Figure 24:**
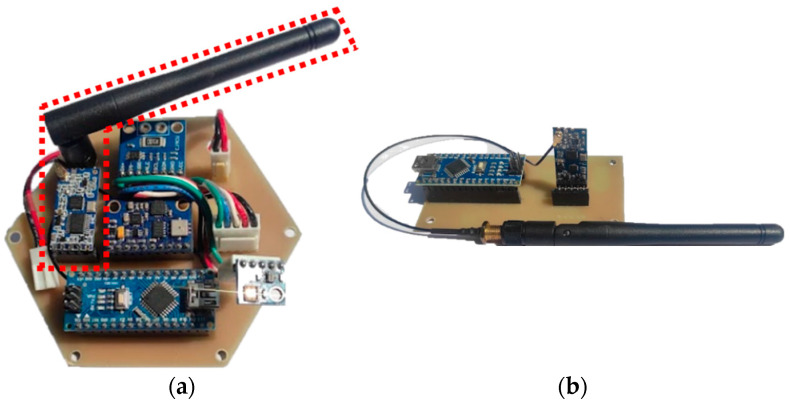
Transmission-reception system; (**a**) transmitter; (**b**) receiver.

**Figure 25:**
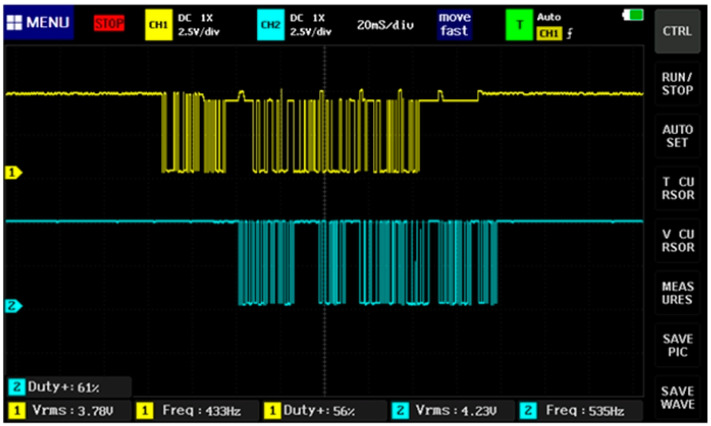
Transmission data in UART communication protocol.

The transmitter and receiver systems are shown in Figure 24a and Figure 24b, respectively. In Figure 25, the data of the communication protocol UART are shown (the signal in yellow corresponds to TX, and the signal in blue to RX). This protocol allows the transmission of data obtained from the sensors through the transmitter and receiver HC-12.

**Figure 26:**
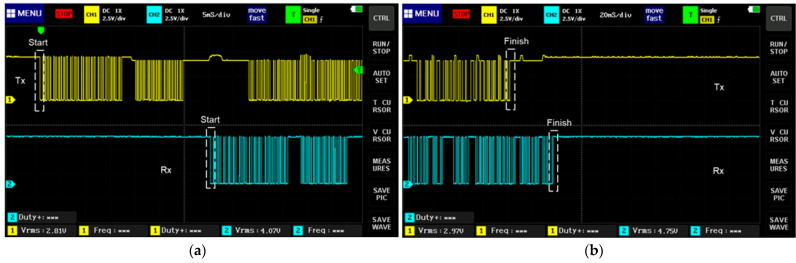
UART protocol. (**a**) Frame start status; (**b**) frame end status.

The communication UART protocol is asynchronous, as it does not share a clock signal; therefore, it has a start bit that changes from a high level to a low level, followed by the data frame, as can be seen in Figure 26a. In the same way, it has an end bit that changes from a low level to a high level, as is shown in Figure 26b.

The transmitter is configured using the AT + CH001 commands for channel 1 with an operating frequency of 433.4 MHz and the AT + P4 command for a transmission power of −8 dBm.

**Figure 27:**
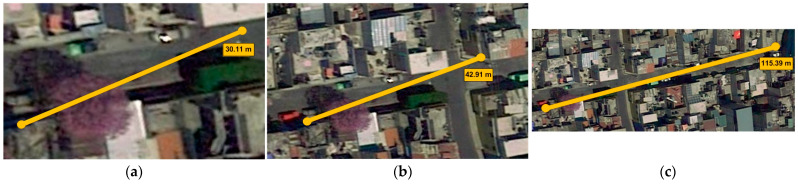
Transmission-reception system evaluation. (**a**) 30 m; (**b**) 43 m; (**c**) 115 m.

To evaluate the performance of the transmission-reception system, it was tested at different distances. The first condition considers 30 m, as shown in Figure 27a. In the second test, 43 m was achieved, as is shown in Figure 27b; the system presented intermittence in the data reception, so the data transmission time was extended from 300 ms to 1 s per reading. This allowed an increase in the transmission distance to 115 m, as is shown in Figure 27c.

#### 4.2.9. Telemetry Interface 

**Figure 28:**
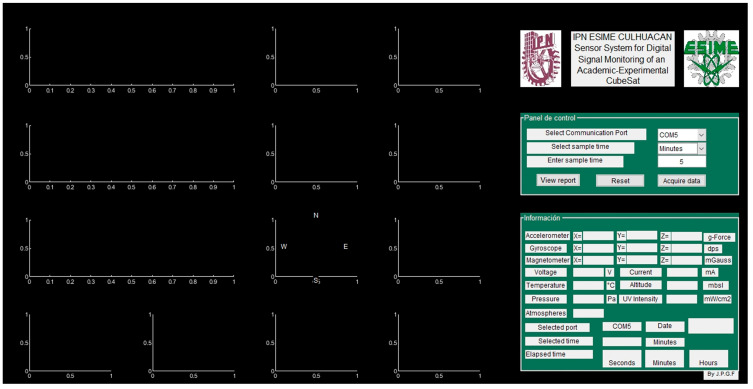
Telemetry interface.

**Figure 29:**
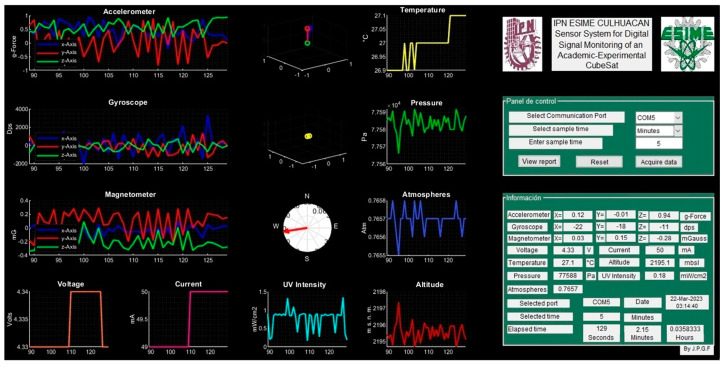
Data acquisition process in the telemetry interface.

**Figure 30:**
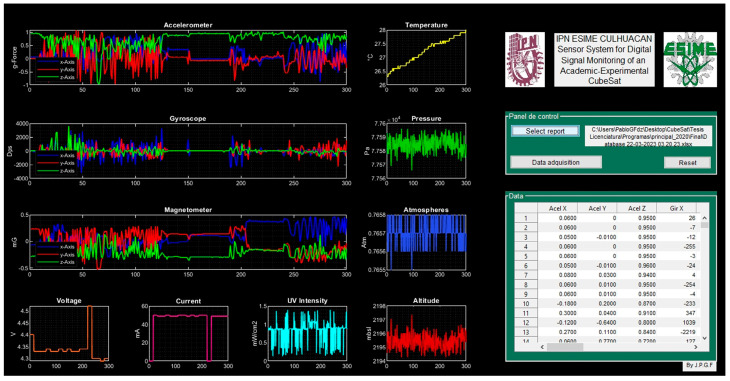
Data visualization in telemetry interface: set (s_1_), set (s_2_), set (s_3_), ser (s_4_), set (s_5_), set (s_6_), and set (s_7_).

The developed telemetry interface is shown in Figure 28; data display fields were defined for each sensor. Figure 29 shows the telemetry interface during the data acquisition process and indicates the acquisition date, the selected port, and the time window selected for each sensor. Once the data acquisition process is completed, the reporting option is selected, and the data acquired by the sensors are loaded, generating their graphical representation, and the values are listed in a table for later analysis, as is shown in Figure 30.

Finally, in Section 2, the sensing set ∑_2_, such as ∑_2_ = {s_1_, s_2_, s_3_, s_4_, s_5_, s_6_, s_7_} is presented. It includes acceleration (s_1_), angular velocity (s_2_), magnetic field (s_3_), temperature (s_4_), pression (s_5_), ultraviolet light intensity (s_6_), and level of battery charge (s_7_); each subset is described, and the results on design and implementation probes are presented. The energizing set: ∑_1_ = {e_1_, e_2_, e_3_, e_4_} obtains the energy from the five panels. The voltage provided by the panels (e_4_) is filtered and regulated by a DC voltage regulator Buck converter (e_3_), which is used as a battery charger (e_2_) and voltage booster (e_1_); it is important to mention that voltage and current values are lower than the data provided by the manufacturer; therefore, measured data are used for design purposes to quantify the power provided by the solar panel to the system; it is caused by environmental conditions like temperature, shading, and dust accumulation; its degradation or aging of the solar panel, measurement inaccuracies are the result of equipment calibration or limitations, or variations in the resistive load during measurements.

## 5. Discussion

A comparison between the design results for the academic–experimental CubeSat for wireless telemetry purposes and similar designs [19,20,21,22,23] is summarized in Table 8. 

Academic–experimental CubeSat provides a significant number of sensors, similar to the academic proposal reported in the literature. 

The processor used in terms of resources is similar to the proposals presented in [19,20,21,22], so it is assumed that the experimental results reported in the article have a similar impact to the proposals listed in Table 8.

## 6. Conclusions

The CubeSat standard is an easy access option to the satellite technology, which is widely used in telecommunications for climate monitoring and scientific research; this satellite type is the case of study in this paper. The authors present a methodology of design of measuring and energizing sensor system (temperature, magnetic fields, pressure, UV rays, position, velocity, and electrical voltage and electrical current) for digital signal monitoring of an academic–experimental CubeSat-1U for wireless telemetry purposes. For this goal, we identify four basic subsystems of the satellite: energization subsystem, sensing subsystem, transmission and reception subsystem, and control subsystem, describing in detail the construction of the first and second subsystems by set diagrams that define their operation and interaction. Also, the design includes basic systems, such as power, control, sensing, transmission, and reception systems. 

For the energizing system, the authors propose a subset ∑_1_ that provides electric energy to the CubeSat subsystems. This energy is obtained from the set of five panels. The voltage provided by the panels (e_4_) is filtered and regulated by a DC voltage regulator Buck converter (e_3_), which is used as a battery charger (e_2_) and voltage booster (e_1_). In this system, voltage and current values are lower than the data provided by the manufacturer; therefore, measured data are used for design purposes to quantify the power provided by the solar panel to the system. It is caused by environmental conditions, its degradation, or aging of the solar panel. Measurement inaccuracies are due to equipment calibration, limitations, or variations in the resistive load. For the monitoring system, we propose a subset ∑_2_ that measures physical parameters such as acceleration (s_1_), angular velocity (s_2_), magnetic field (s_3_), temperature (s_4_), pression (s_5_), ultraviolet light intensity (s_6_), and level of battery charge (s_7_). Each subsystem was presented in the paper, including its design and experimental results. 

Finally, as contribution and key aspects of this research, the methodology of design, integration, and development of the four subsystems were presented, corroborating the feasibility of construction and its implementation in an academic satellite CubeSat. For this purpose, each subsystem was detailed as a set of elements represented through diagrams showing the interaction of each element and its function. In addition, the design and integration methodology of the entire measurement and sensor energization system of the CubeSat satellite was presented, emphasizing the use of commercial and easily replaceable components.

## Figures and Tables

**Table 1:** Energy consumption of sensors.

Sensor	Voltage/Current
Microcontroller Atmega328P of Microchip	5 V/50 mA
Accelerometer ADXL345 of Analog Devices	3.6 V/140 µA
Pressure sensor BMP085 of Bosh	3.6 V/12 µA
Gyroscope L3G4200D of STM	3.6 V/1.5 mA
Magnetometer HMC5883L of Honeywell	3.6 V/100 mA
Current sensor INA226 of Texas Instrument	5.5 V/10 mA
Sensor of intensity of UV of LAPIS	4.6 V/0.5 mA
Transmitter HC-12 of STM	5.5 V/200 mA

**Table 2:** Current and power generation process.

Steps	Value
Step 1: Current_LSB_	31 × 10^−6^
Step 2: Calibration register	1652
Step 3: Current register	32,266
Step 4: BusVoltage register	3920
Step 5: Power register	6324

Where in step 1, the maximum expected current is 1 A, in step 2, *R_shunt_* = 0.1 Ω, and in step 3, the voltage through *R_shunt_* must be divided by the internal step size of the sensor 2.5 µ.

**Table 3:** I²C address.

Parameter Sensed	Sensor Model	I²C Address
Acceleration	ADXL345	0 × 53
Angular Velocity	L3G4200D	0 × 69
Magnetic Field	HMC5883L	0 × 1E
Pressure and Temperature	BMP085	0 × 77

**Table 4:** AT commands for HC-12 transmitter-receiver.

Command	Description
AT	Test command
AT + Bxxxx	Command to change the UART transmission speed.
AT + Cxxx	Command to change the communication channel.
AT + Fux	Command to change transmission mode FU1, FU2, FU3, FU4.
AT + Px	Prepare the transmission power (dBm).
AT + Ry	Command to request configuration information for each parameter.
AT + Rx	Get the current configuration values.
AT + Uxxx	Command to enable or disable parity control.
AT + V	Shows the firmware version and website.
AT + SLEEP	Command to start power-saving mode (no data transmission).
AT + DEFAULT	Restores the factory values.

**Table 5:** Comparison of parameters in solar panel.

Parameter	Experimental Measurement	Manufacturer Data
Open circuit voltage	6.4 V	7.0 V
Short circuit current	153.3 mA	203 mA
Nominal voltage	5.24 V	5.5 V
Nominal current	132.19 mA	150 mA
Nominal power	0.69 W	0.85 W

**Table 6:** Output voltages in the Buck converter.

Panels Covered by Shadow	Measurement
0	4.6 V
1	4.5 V
2	4.4 V
3	4.3 V
4	3.6 V
5	3.6 V

**Table 7:** Battery output voltage.

Panels Covered by Shadow	Measurement
0	4.1 V
1	4.1 V
2	4.1 V
3	4.1 V
4	4.1 V
5	3.9 V

**Table 8:** Comparison of prototype.

	Target/Purpose	Sensors	Processor
Proposed	Academic	Voltage, current, accelerometer, gyroscope, magnetometer, temperature, barometer altitude, UV	Atmega328P
[19]	Experimental	Magnetometer, inertial measurement, light, motor speed	Raspberry pi
[20]	Academic	Telemetry, magnetometer, light, gyroscope, particle detector	PIC16F877
[21]	Academic study	Image sensors	STM32F407
[22]	Mission analysis	SWIR spectrometer, RGB images, current, battery, temperature	400 MHz ARM9
[23]	In-orbit characterization	Hyperspectral imager (HSI)	FPGA

## Data Availability

The data obtained in this study are available on request from the authors.
